# A Community-Based Survey on Health-Care Utilization for Pneumonia in Children in Peri-Urban Slums of Karachi, Pakistan

**DOI:** 10.4269/ajtmh.18-0656

**Published:** 2019-09-03

**Authors:** Salima Kerai, Imran Nisar, Ilyas Muhammad, Sana Qaisar, Khalid Feroz, Azhar Raza, Faizan Khalid, Benazir Baloch, Fyezah Jehan

**Affiliations:** Department of Pediatrics and Child Health, Aga Khan University, Karachi, Pakistan

## Abstract

Pneumonia, as defined by WHO, is a syndromic diagnosis characterized by presence of cough or difficult breathing. Presentation to health-care provider depends on timely identification of signs and symptoms by caretakers. We explored patterns of health-care utilization among caretakers of a randomly selected sample of 1,152 children aged 2–59 months, residing in low-income settlements of Karachi, Pakistan. Information on household demographics, occurrence of pneumonia-specific symptoms, care seeking, air quality, and knowledge regarding preventive measures for pneumonia was collected. Predictors of care seeking were estimated using weighted logistic regression. Prevalence of pneumonia with cough and rapid or difficulty in breathing was found to be 40.8% and 37.1% in infants (2–11 months) and children (12–59 months), respectively. Ninety-five percentage of caretakers sought care, 68.5% privately. Odds ratios (ORs) for independent predictors of care-seeking were as follows: younger age of child (infants compared with children), 3.60 (95% CI = 2.65–4.87); caretaker with primary education compared with none, 3.40 (2.46–4.70); vaccine awareness, 1.65 (1.45–1.87); and breastfeeding awareness, 1.32 (1.13–1.53). Presence of symptoms such as fever OR, 1.51 (1.30–1.76); tachypnea, 1.57 (1.35–1.83); chest indrawing, 2.56 (2.05–3.18); persistent vomiting, 1.69 (1.37–2.09); and recurrent illness, 2.57 (2.23–2.97) were also predictive. There is high health-care utilization for pneumonia with the skewed presentation toward private services. Strategies should be focused on making pneumonia care standardized, efficient and affordable, especially in the private sector.

## INTRODUCTION

WHO classifies pneumonia based on severity into fast breathing pneumonia, chest indrawing pneumonia and severe pneumonia with general danger signs (stridor when calm, hypoxemia defined as SaO_2_ < 90% in air, inability to feed, vomiting everything, convulsions or reduced conscious level).^1^ Fast breathing pneumonia is a milder form of illness and characterized by isolated fast breathing that can be effectively managed at community level.^1^ According to WHO and United Nations Children’s Fund global estimates such as Multiple Indicator Cluster Surveys (MICS) and Demographic and Health Surveys (DHS), nearly half of caretakers of children in developing countries with symptoms of pneumonia do not seek care.^2,3^ In South Asia alone, one in three children under five with suspected pneumonia do not have access to appropriate health-care provider or facility.^2^

Karachi is the largest urban city of Pakistan, with a burgeoning population of 27.5 million people. Health care is fragmented, comprising a large private sector, including secondary and tertiary hospitals and neighborhood clinics run by general medical practitioners. Alongside function a multitude of unregulated and informal health services such as pharmacies, homeopaths, hakeems, traditional and spiritual healers, and quacks.^4^ One of the largest towns of Karachi district, Korangi town, is the setting for an antibiotic management trial of fast breathing pneumonia in children in four contiguously located communities (Clinical Trials Register NCT02372461).^5^ This article describes the burden of pneumonia among children living in these communities, knowledge of caretakers regarding its diagnosis, prevention, patterns of health-care utilization, and care seeking during pneumonia season. Information about health-care utilization pattern and associated factors will guide resource allocation and improve service delivery to achieve universal health coverage.

## METHODS

### Study setting.

**Figure 1.**
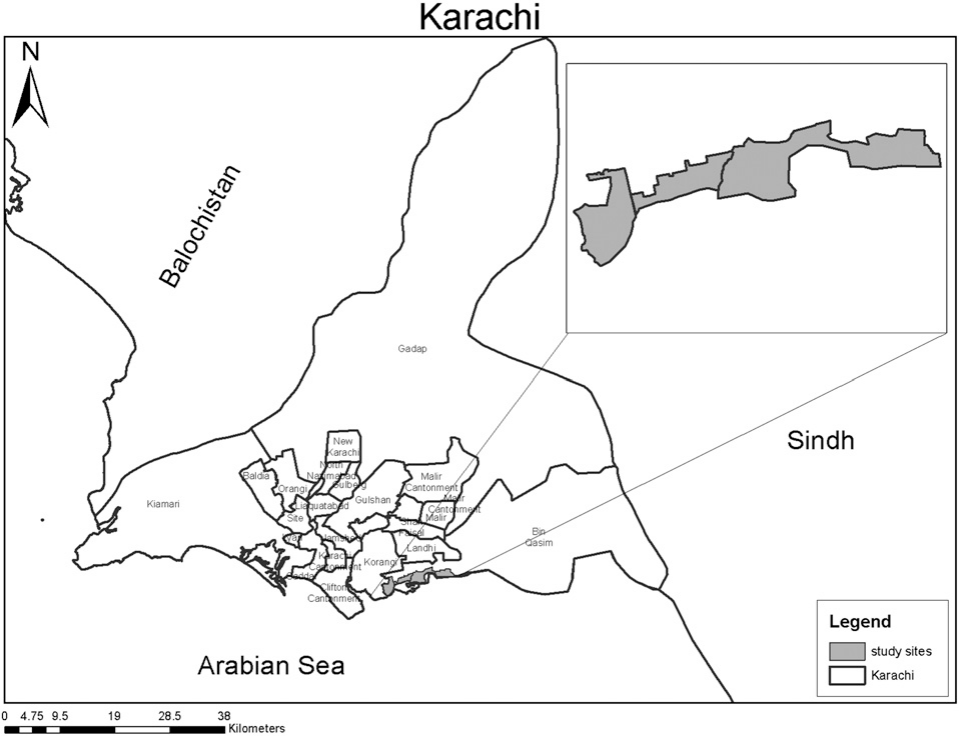
Study sites.

This survey was conducted between March–May 2016 in peri-urban slums located at the outskirts of Karachi along the belt of the Arabian Sea (Figure 1). These low-income settlements are an unplanned urban sprawl accommodating people of various ethnicities but are dominated by Sindhi-speaking Muslims. Communities mostly live in extended families inhabiting shacks and open brick houses along with their livestock and pets. Water supply is from municipal mains through piped water and from community tanks where water is purchased. Waste disposal system is inadequate with open and overflowing sewers. Numerous pharmacies, licensed and unlicensed physicians, and traditional healers operate in this setting. The climate is arid with mostly summer season except from December to February when winter is experienced and monsoons from July to August. Here, Aga Khan University’s Department of Pediatrics and Child Health has maintained demographic surveillance since the past decade along with primary health-care centers (PHCs) for management of common childhood illnesses. According to the last survey (2010), the total population is 198,494. There are 28,000 children younger than 5 years, under-5 mortality is 76/1,000 live births, HIV seroprevalence is < 0.1%, and malaria is endemic with low transmission.^6^ In a previous survey, pneumonia incidence was reported to be 25 cases per 100,000 child-years with a case fatality rate of 1.5%.^7^ The PHCs located in this area are staffed by trained physicians who offer free services for improving child health. Children younger than 5 years are self-referred here by caretakers.

### Case definition and study population.

For the purpose of this study, suspected fast breathing pneumonia is defined as children with cough accompanied by self-reported rapid or difficulty in breathing. Caretakers of children 2 months to 5 years of age residing in these communities for at least 1 year were included. To assess the period prevalence of pneumonia, a reference period of 8 weeks caretaker recall of symptoms and signs was used. Previously conducted household surveys (MICS question and DHS) that produce coverage estimates in low-income countries have used 2-week recall for monitoring the proportion of children with pneumonia.^8,9^ Critical review of these surveys have indicated that discriminatory power of the tool can be increased by performing the survey in peak pneumonia season.^10^ Also, we may be able to capture an upper estimate of prevalence by increasing the recall period (Shamim Qazi, personal communication). Therefore, we performed the survey between March and May to cover a recall period of the last 8 weeks in the pneumonia season.

### Data collection.

Data were gathered on precoded standardized questionnaire by trained community health-care workers (CHWs). Community health-care workers sequentially visited the households of children randomly selected from a sampling frame provided by surveillance system and administered the questionnaire. The primary respondent was the main caretaker or the mother. If the caretaker or the mother was absent, or the house was locked, three attempts were made to establish contact. In case contact could not be established or there was refusal to participate, the next child on the list was contacted. Information on household demographics and composition, occurrence of pneumonia, vaccination, air quality, and knowledge regarding preventive measures for pneumonia was collected. If caretaker recalled an incident of pneumonia in past 8 weeks, further questions were asked regarding specific symptoms such as fever, fast breathing, chest indrawing, and danger signs. Also, questions were asked regarding care seeking, use of drugs, outcome, and hospitalization. If the child did not experience the disease or related symptoms in the past 8 weeks, hypothetical questions were asked about anticipated health-care use in case the child developed such an illness.

### Sample size and statistical analysis.

Using previous estimates of pneumonia prevalence from the same community (15% for infants and 10% for children)^7^ with estimated care seeking to PHC as 30% for both age categories,^11^ 10% precision, and 5% alpha, a sample of 540 infants and 810 children was required. The sample size was stratified on age because of differences in disease occurrence and health seeking pattern between younger and older children.

To examine distribution and data, descriptive statistics were computed. Mean and SDs for normal distribution and median and interquartile ranges for skewed distribution were reported. Frequency and percentage was computed for categorical variables. Eight-week period prevalence of fast breathing pneumonia was calculated. Predictors of health-care utilization for children with cough were determined using weighted logistic regression. Weights were assigned to each infant or child based on the population age structure from prior surveillance. The weight for each responder is the population total for that age category divided by number of children who participated in the survey in that age category.

Purposeful selection model building approach was used. All clinical and statistically significant (having a *P*-value of 0.2 or less) variables, at the univariate level, were considered for the multivariate model. For inclusion in the final model, a *P*-value of less than 0.05 was considered to be significant. All analyses were performed using STATA version 12 (StataCorp., College Station, TX).

## RESULTS

### Demographic profile of all households.

**Figure 2.**
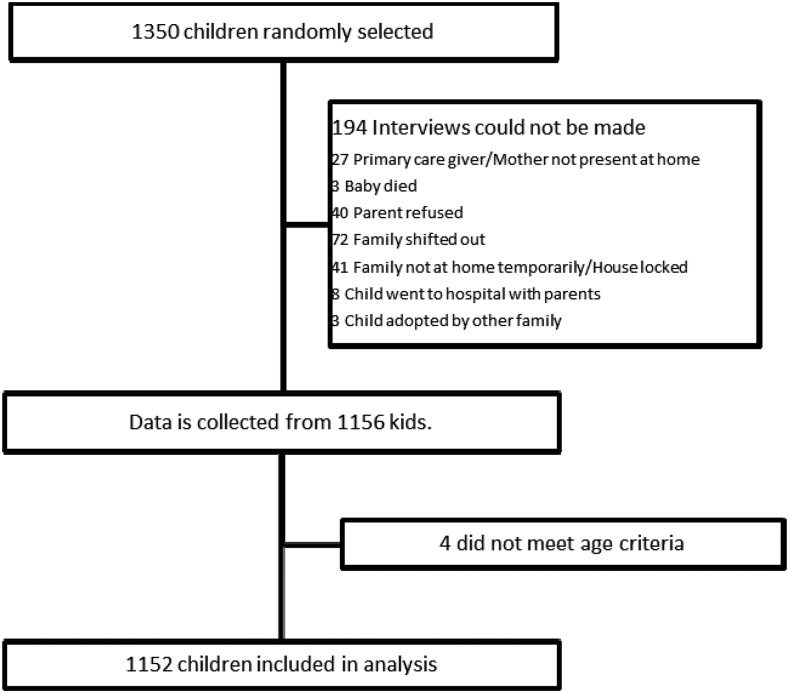
Flow diagram.

Information on 1,152 children was collected (Figure 2, Table 1). Males and females were equally distributed (47.2% males in infants, 52.8% in children) (see Table 1). Median household size was eight (IQR 5–10), with a median of two children (IQR 1–2) younger than 5 years; 66.2% households were living under USD 5 a day. Fishing comprised 33.3% of the livelihood followed by staff jobs (34.6%) and skilled labor (18.5%); 99.9% of the primary caretakers were mothers and 59.2% had never been to school. Among age-eligible children (≥ 3.5 months) using both verbal and card reporting, combined Diphtheria–Pertussis–Tetanus–Hemophilus Influenza B–Hepatitis B vaccine coverage for three doses was 64.4% for infants and 73.6% for children. Pneumococcal vaccine (PCV) coverage for three doses was 52.4% and 43.0%, respectively; 2.4% of infants and 6% of children reported a history of asthma, whereas allergies (food and others) were reported by 8.2% and 10% of caretakers of infants and children, respectively; and 56.4% of the caretakers recognized vaccination to be preventive for pneumonia. Only 37.3% knew that handwashing and 29.7% knew that exclusive breast feeding was preventive. For air quality–related practices, 74.3% of the households opened windows regularly for ventilation and 31.6% had a current smoker in their household. Almost 13% of households used wood as a source of fuel and open fire for cooking, 57% of households were located near open garbage, and 48% near an open sewer.

**Table 1:** Demographic profile of all households

	Total (*N* = 29,996)	Infants (*N* = 4,059)	Children (*N* = 25,937)
Household size (median, IQR)	8 (5–10)	7 (6–11)	8 (5–10)
Number of children in each house (median, IQR)	4 (2–5)	4 (2–5)	4 (2–6)
Number of children under five (median, IQR)	2 (1–2)	2 (1–3)	2 (1–2)
Gender			
Male	50.4%	47.2%	50.9%
Female	49.6%	52.8%	49.1%
Education of primary caretaker			
Never been to school	59.2%	54.3%	59.9%
Up to Primary	20.2%	22.8%	19.8%
Matriculation	17.3%	19.5%	17.0%
Above Matric	3.3%	3.3%	3.3%
Occupation of the head of household			
Fishing	33.3%	30.4%	33.8%
Professional service/staff job/business	34.6%	35.9%	34.4%
Skilled labor (driver, electrician, plumber, carpenter, craftsmen, etc.)	18.5%	21.1%	18.1%
Unskilled labor (domestic worker, street vendor, waste picker, etc.)	12.6%	12.6%	12.6%
Unemployed	1.0%	0.0%	1.1%
Average household income	66.2%	67.8%	65.9%
≤ 5 USD per day	33.8%	32.2%	34.1%
> 5 USD per day			
Vaccination record, *N* = 29,573 (children ≥ 3.5 months of age)			
Ever vaccinated	84.1%	82.7%	84.3%
Verbal	78.9%	60.5%	81.4%
Card verified	21.1%	39.5%	18.6%
Vaccination history			
BCG	83.0%	100.0%	98.5%
Pentavalent 1	75.6%	91.6%	89.7%
Pentavalent 2	68.6%	77.5%	82.1%
Pentavalent 3	61.0%	64.4%	73.6%
PCV 1	42.6%	73.4%	47.5%
PCV 2	40.1%	62.6%	45.7%
PCV 3	37.1%	52.4%	43.0%
Measles I	40.4%	14.4%	52.6%
Child has history of asthma			
Yes	5.5%	2.4%	6.0%
No	94.5%	97.6%	94.0%
Child has any known allergies			
Yes	9.7%	8.2%	10.0%
No	90.3%	91.8%	90.0%
Knowledge about ways to prevent pneumonia? (multiple response question)			
Vaccination	56.4%	58.8%	56.1%
Reducing indoor air pollution	30.7%	28.2%	31.1%
Exclusive breast feeding	29.7%	31.9%	29.4%
Handwashing	37.3%	40.2%	36.8%
Adequate nutrition	18.8%	18.0%	19.0%
None of the above method	6.5%	4.9%	6.7%
Home remedies	7.7%	10.2%	7.3%
Regularly open windows of household for proper ventilation	74.3%	77.0%	73.9%
Houses with presence of pets/birds with fur/feathers in your household	26.7%	27.1%	26.7%
Current smoker in your household	31.6%	32.2%	31.5%
Type of fuel used in household for cooking			
Wood	12.4%	11.8%	12.6%
Biofuel/animal dung	1.1%	0.7%	1.1%
Kerosene oil	0.6%	0.0%	0.7%
Coal	0.0%	0.0%	0.0%
Natural gas	85.9%	87.6%	85.6%
Type of cooking stove used			
Open fire	13.5%	12.4%	13.7%
Close stove	86.5%	87.6%	86.3%
Place of cooking			
In a room used for living/sleeping	6.9%	6.9%	6.8%
In a separate room used as kitchen	58.9%	57.0%	59.2%
a separate building used as kitchen	28.8%	28.4%	28.8%
Outdoors/Sehan	5.5%	7.8%	5.1%
Child usually present near the cooking area while you are cooking	33.7%	26.8%	34.8%
Household location (multiple response question)			
Factory/power plant	12.6%	14.9%	12.3%
Public oven/furnace	3.3%	3.5%	3.3%
Traffic fumes	20.8%	19.7%	21.0%
Rubbish pile/garbage lot	57.2%	60.1%	56.8%
Sewer	48.2%	54.1%	47.2%

PCV = pneumococcal vaccine.

### Pneumonia prevalence and symptoms.

Fever and fast breathing as perceived by caretaker were the most common symptoms reported in children (85.8% in infants and 57.8% in children) (Table 2). Chest indrawing was reported by 33.9% of infants and 28.9% of children. Feeding problems were reported more in children (24.4%), whereas vomiting was reported in infants (29.6%). Lethargy or unconsciousness was reported to be 11.3% and 9.3%, respectively. The 8-week period prevalence of suspected fast breathing pneumonia among children with cough, who have rapid or difficulty in breathing, was found to be 40.8% and 37.1% in infants and children, respectively, and 36.2% of infants and 39% of children had at least two episodes of fast breathing pneumonia in the last 2 months. The duration of illness was almost similar in both infants and children (median of 5 (IQR 3–7) days and 4 (IQR 3–7), days respectively).

**Table 2:** Symptoms in children with pneumonia

	Total (*N* = 19,507)	Infants (*N* = 2,709)	Children (*N* = 16,798)
Symptoms during illness			
Fever	85.8%	89.0%	85.2%
Tachypnea or difficulty in breathing	57.8%	61.1%	57.3%
Chest indrawing	29.6%	33.9%	28.9%
Convulsions	1.7%	4.0%	1.3%
Lethargic or unconsciousness	9.5%	11.3%	9.3%
Feeding problem	24.1%	21.9%	24.4%
Vomiting	24.8%	29.6%	24.0%
Hypoxia	1.4%	3.3%	1.1%
Frequency of episodes with suspected pneumonia			
1 Episode	31.8%	26.6%	32.6%
2 Episode	38.6%	36.2%	39.0%
More than two episodes	29.6%	37.2%	28.4%
Duration of illness in days (median, IQR)	4 (3–7)	5 (3–7)	4 (3–7)

### Health-care seeking behavior.

Overall, 95% of caretakers sought care and 68.5% went to private health-care providers. Children who came to PHCs were 27.8% of infants and 16.9% of children (Table 3). Primary health-care center utilization for both fast breathing and chest indrawing or danger signs was higher in infants than in children (27.6% and 29% versus 12% and 13.6%). Overall, private care was preferred for children for all pneumonia. Utilization of public sector health services was low (11% and 10% for fast breathing and chest indrawing or danger signs). About 5% of caretakers did not seek any care, 3% of whom had chest indrawing or danger signs. Reasons identified were clinic too far from home, siblings at home that could not be left alone, and high cost of treatment. Of these, 19% of children were self-medicated. None of the infants were self-medicated.

**Table 3:** Health seeking behavior for all children as well as by severity of pneumonia

Health seeking behavior	All Children			Children with fast breathing pneumonia			Children with severe pneumonia (chest indrawing pneumonia and/or danger sign)		
	Weighted Total	Infants aged 2–11 months	Children aged 12–59 months	Weighted total	Infants aged 2–11 months	Children aged 12–59 months	Weighted Total	Infants aged 2–11 months	Children aged 12–59 months
	*N* = 29,996	*N* = 4,059	*N* = 25,937	*N* = 11,276	*N* = 1,656	*N* = 9,620	*N* = 10,225	*N* = 1,530	*N* = 8,695
Seek medical care for child outside your home	95.0%	97.8%	93.7%	99.4%	98.4%	96.5%	97.4%	99.4%	97.0%
Pharmacy/medical store	1.2%	1.1%	1.2%	0.0%	0.0%	1.2%	0.7%	0.0%	0.9%
Friend/relative/traditional healer/faith healer	0.6%	0.2%	0.6%	0.0%	0.6%	0.8%	0.4%	0.0%	0.4%
Aga Khan University primary health-care center	18.5%	27.8%	16.9%	29.0%	27.6%	12.0%	15.9%	29.0%	13.6%
Charitable/philanthropic hospital	3.1%	3.6%	3.0%	4.7%	2.8%	2.0%	2.6%	4.7%	2.2%
Private clinic/hospital	68.5%	59.0%	70.0%	55.0%	58.6%	72.1%	70.1%	55.0%	72.8%
Government clinic/hospital	8.2%	8.1%	8.2%	11.2%	10.5%	12.0%	10.3%	11.2%	10.1%
Do not seek medical care outside home	5.0%	2.2%	6.3%	0.6%	1.6%	3.5%	2.6%	0.6%	3.0%
Clinic too far from house	31.3%	55.6%	30.0%	0.0%	66.7%	55.6%	0.0%	0.0%	0.0%
Unable to find transport/cost for travel too high	14.8%	11.1%	15.0%	0.0%	0.0%	11.1%	27.6%	0.0%	28.6%
Cost for treatment too high	17.7%	22.2%	17.5%	0.0%	0.0%	11.1%	41.4%	0.0%	42.9%
Children at home who could not be left alone	17.2%	11.1%	17.5%	100.0%	33.3%	11.1%	17.2%	100.0%	14.3%
Bought medicine on their own	19.0%	0.0%	20.0%	0.0%	0.0%	11.1%	13.8%	0.0%	14.3%

### Management of pneumonia.

Ninety percentage of children received antibiotics during treatment (80% orally and 20% parenterally) (Table 4). The majority of children (96%) improved with treatment and 2% got hospitalized. Most hospitalizations were in public sector hospitals.

**Table 4:** Management of children with pneumonia

	Total (*N* = 19,507)	Infants aged 2–11 months (*N* = 2,709)	Children aged 12–59 months (*N* = 16,798)
Children received antibiotics during treatment	90.4%	89.7%	83.5%
Parenteral	20.2%	19.3%	20.3%
Oral	79.8%	80.7%	79.7%
Outcome of treatment			
Improved	96.5%	96.7%	96.5%
Worsen	0.2%	0.3%	0.2%
Unchanged	3.3%	3.0%	3.3%
Children hospitalized for more than 24 hours	2.5%	4.0%	2.2%
Charitable/philanthropic hospital	7.7%	0.0%	10.0%
Private clinic/hospital	21.1%	25.0%	20.0%
Government clinic/hospital	71.1%	75.0%	70.0%

### Predictors of care seeking.

Predictors of care seeking for pneumonia were infancy (odds ratio (OR) 3.6; 95% CI: 2.65, 4.87), caretaker with primary education (OR 3.4; 95% CI: 2.46, 4.70), and income greater than USD 5 (OR 1.14; 95% CI: 1.00, 1.31) (Table 5). Other predictors were fever (OR 1.57; 95% CI: 1.35, 1.83), tachypnea (OR 1.52; 95% CI: 1.31, 1.76), chest indrawing (OR 2.56; 95% CI: 2.05, 3.18), and persistent vomiting (OR 1.69; 95% CI: 1.37, 2.09). Unusually, presence of lethargy or unconsciousness negatively predicted care seeking (OR 0.54; 95% CI: 0.41–0.72). In addition, caretakers whose children have recurrent pneumonia episodes were more likely to seek care (OR 2.57; 95% CI: 2.23, 2.97). Also, caretakers whose children were completely vaccinated for pentavalent and PCV (OR 1.28; 95% CI: 1.12, 1.46) and who were aware about vaccination (OR 1.65; 95% CI: 1.45, 1.87) or were aware about the role of breastfeeding in prevention of pneumonia (OR 1.32; 95% CI: 1.13, 1.53) were more likely to seek care.

**Table 5:** Predictors of health-care utilization (seeking care vs. no care) for all children

	Adjusted OR	95% CI
Age		
Infant	3.6	2.65.4.87
Child	1	
Gender		
Female	1.33	1.18.1.51
Male	1	
Education of caretaker		
Never been to school	1.24	0.94.1.63
Up to Primary	3.4	2.46.4.70
Up to secondary	1.83	1.35.2.49
Intermediate and above	1	
Income		
≤ 5 USD per day	1	
> 5 USD per day	1.14	1.00.1.31
Fever		
No	1	
Yes	1.57	1.35.1.83
Tachypnea or difficulty in breathing		
No	1	
Yes	1.52	1.31.1.76
Chest indrawing		
No	1	
Yes	2.56	2.05.3.18
Lethargy or unconsciousness		
No	1	
Yes	0.54	0.41.0.72
Vomits everything		
No	1	
Yes	1.69	1.37.2.09
Frequency of suspected pneumonia episodes		
1 episode	1	
2 episodes	2.57	2.23.2.97
> 2 episodes	2.73	2.26.3.29
Completely vaccinated (three doses of Hib and pneumococcal vaccine )		
No	1	
Yes	1.28	1.12.1.46
Awareness about vaccination to prevent pneumonia		
No	1	
Yes	1.65	1.45.1.87
Awareness about breastfeeding to prevent pneumonia		
No	1	
Yes	1.32	1.13.1.53
Number of observations	21,368	
McFadden’s *R*^2^	12%	

## DISCUSSION

The study findings indicated high health-care utilization for pneumonia in this peri-urban setting. However, awareness about handwashing, breastfeeding, and particularly the role of vaccination in pneumonia prevention remained low. Studies from Pakistan and other developing countries showed that caregiver associate pneumonia with factors other than infection such as cold air, implying etiology of pneumonia is not well understood by community.^12–15^

For outpatient treatment, there was a preference for private out-of-pocket treatment or treatment at primary health-care center. This utilization pattern is not different from previous surveys reported from a similar setting in Pakistan.^11,12^ Public-sector health facilities were underutilized, especially for outpatient treatment; however, they were used mostly in case of hospitalization. Possible reasons for this are non-availability of qualified staff and medicines, and compromised quality of care.^4,16,17^ This was also seen in the Karachi, Sindh MICs survey of 2014 that reported public service utilization of 18.7% for acute respiratory infections.^8^ This pattern of utilization is not unique to South Asia or other developing countries, where most outpatient visits occur in private sector.^17–19^ For example, 92% of children in Guatemala and more than 70% in Egypt sought care for pneumonia from private clinics.^20,21^

In this area, there has been mushroom growth of private and informal health-care facilities in recent years. Such providers adhere less to practice guidelines for diagnosis and treatment.^22,23^ Irrespective of socioeconomic status, caretakers are willing to pay out of pocket for private and informal health services. Factors such as client-perceived quality of care, responsiveness, and geographical access may play an important role in determining the kind of service and facility used.^18^ This is a potential area for further investigation to inform interventions for improving health seeking and care for pneumonia in children.

Primary health-care center utilization was higher in case of infants. The surveillance is primarily focused around pregnant women and newborns, and this may be the reason for the PHC seeing more infants. Age less than 12 months was also found to be an independent predictor of care seeking. Care seeking behavior for children with suspected pneumonia in sub-Saharan African countries showed that the youngest children (< 2 years) were more likely to be brought to a care provider in Nigeria, Ethiopia, and the Democratic Republic of the Congo.^24^ Young age is a critical time where the incidence and mortality is the highest for the illness.^7^

Other caretaker factors influencing care seeking were caretaker having income greater than USD 5 a day. Of note, Aga Khan University PHC and other public health facilities running in these communities are operating free of cost. Intuitively direct cost of medical care should not deter caretakers from seeking care. However, there are substantial nonmedical costs that are unaccounted for, for instance, the cost of transportation to a health-care facility, time taken off from home or work to go to the health facility, the cost of suffering and physical discomfort, cost of food and place of stay near heath facility, etc.^25^

The recognition of the child’s illness by the mother should have a major impact on health seeking behaviors.^25^ In the present study, chest indrawing as recognized by the mother, showed the highest association with care seeking, whereas recognition of fever, tachypnea, or vomiting showed a smaller but positive influence. This is not validated with CHW or health-care provider identification and based only on maternal perception and recall. Validated assessments from Bangladesh indicated that maternal recognition of these signs was low, where only one of four mothers could correctly identify symptoms such as convulsion, unconsciousness, chest indrawing, unable to feed, etc., when compared with CHW.^26^

There are several strengths of our study. The household survey has used a random sample of respondents in a large peri-urban community with a low refusal rate. Hence, study results represent care seeking of children with suspected pneumonia from low-income urban slums in Pakistan. Caretaker history for identification of pneumonia episodes was obtained using the standard MICS and DHS survey strategies. Their methodology was improvised by increasing the period of recall to identify more cases and improve specificity by conducting the survey to cover the peak pneumonia season. This is the first report of utilization of such a strategy. One may presume improved discriminatory power of the MICS and DHS questions and is a potential area of further study. Although not the primary intent, pneumonia prevalence was also calculated using 8-week period prevalence of pneumonia. This survey is the latest since the last MICS survey of Sindh 2014,^8^ focusing on an impoverished urban community.

There are some limitations in the present study, such as caretaker recall bias that may underestimate true disease. Conducting the survey in peak pneumonia season is also the season when upper respiratory illnesses are more incident, which may have led to over-reporting of respiratory symptoms. Reasons for specific health-care utilization pattern were not explored in a qualitative analysis framework. Further exploratory studies are required to establish the determinants of choice of care.

## CONCLUSION

At least 38.6% of children have two episodes of pneumonia over 8 weeks, based on caretaker recall. More than 90% of caretakers seek health-care with utilization skewed toward regulated or unregulated private services. For hospitalizations, the preference is toward public sector facilities. Targeting private services for standardizing outpatient pneumonia care, especially for fast breathing pneumonia, can lead to improved diagnosis and treatment and prevention of unnecessary antibiotics use. Also, improved awareness for vaccination and education to detect signs of pneumonia can result in better health-care seeking at the community level.
